# Temperature Sensitive Photosynthesis: Point Mutated CEF-G, PRK, or PsbO Act as Temperature-Controlled Switches for Essential Photosynthetic Processes

**DOI:** 10.3389/fpls.2020.562985

**Published:** 2020-09-25

**Authors:** Vinzenz Bayro-Kaiser, Nathan Nelson

**Affiliations:** Department of Biochemistry, The George S. Wise Faculty of Life Sciences, Tel Aviv University, Tel Aviv, Israel

**Keywords:** photosynthesis, temperature-sensitive photosynthesis, temperature-sensitive mutants, Chloroplast-Elongation-Factor-G, Phosphoribulokinase, photosystem II, PsbO, *Chlamydomonas reinhardtii*

## Abstract

Temperature sensitive mutants have been widely used to study structure, biogenesis and function of a large variety of essential proteins. However, this method has not yet been exploited for the study of photosynthesis. We used negative selection to isolate temperature-sensitive-photoautotrophic (TSP) mutants in *Chlamydomonas reinhardtii*. From a population of randomly mutagenized cells (n=12,000), a significant number of TSP mutants (n=157) were isolated. They were able to grow photoautotrophically at 25°C, but lacked this ability at 37°C. Further phenotypic characterization of these mutants enabled the identification of three unique and highly interesting mutant strains. Following, the selected strains were genetically characterized by extensive crossing and whole genome sequencing. Correspondingly, the single amino acid changes P628F in the Chloroplast-Elongation-Factor-G (CEF-G), P129L in Phosphoribulokinase (PRK), and P101H in an essential subunit of Photosystem II (PsbO) were identified. These key changes alter the proteins in such way that they were functional at the permissive temperature, however, defective at the restrictive temperature. These mutants are presented here as superb and novel tools for the study of a wide range of aspects relevant to photosynthesis research, tackling three distinct and crucial photosynthetic processes: Chloroplast translation, PET-chain, and CBB-cycle.

## Introduction

Photosynthesis is one of the most important reactions on planet earth. It provides the necessary energy and organic building blocks for almost all living beings and it may be critical for the development of sustainable fuel production. Therefore, understanding and engineering photosynthesis is crucial in order to address humanity’s major challenges of increasing food production and deploying clean and sustainable energy production. Most photosynthetic organisms perform oxygenic photosynthesis. It is a very complex process which requires the tight coordination of a large variety of active components. It can be divided in two major steps: the light reactions on the thylakoid membranes and the carbon fixation reactions in the stroma (Taiz and Zeiger, 2002).

The light reactions consist of converting light energy into chemical energy. It comprises protein complexes embedded in the thylakoid membranes, soluble proteins in the lumen and the stroma, and a pool of mobile quinone molecules in the membranes. The membrane protein complexes are photosystem II (PSII), cytb_6_f-complex, photosystem I (PSI), and ATP-synthase (ATPase). The luminal proteins are Plastocyanin or soluble cytochrome. The stromal proteins are ferredoxin and ferredoxin-NADP+-reductase (FNR). The reaction centers of PSII and PSI are responsible for converting absorbed light energy into redox potential. This redox potential drives a chain of redox reactions, the photosynthetic electron transport chain (PET chain). The PET chain oxidizes water in the lumen and transfers the obtained electrons through PSII, plastoquinone, cytb6f-complex, plastocyanin, and PSI to reduce ferredoxin in the stroma. Concomitantly, a proton gradient is generated along the thylakoid membranes. Reduced ferredoxin can provide electrons to FNR to reduce NADP+ to NADPH. While the proton gradient drives the ATP synthase to phosphorylate ADP to ATP. The thylakoid reactions result in O_2_, ATP, and NADPH production.

The carbon fixation reactions consist of a cycle of enzymatic reactions called the Calvin-Benson-Bassham-cycle (CBB-cycle) (Taiz and Zeiger, 2002). It can be divided in three phases. The carbon fixation phase is composed by a single reaction where CO_2_ is fixed into ribulose-1,5-bisphosphate (RuBP) to generate 3-phosphoglycerate (3-PGA). The reduction phase consists of two reactions that use ATP and NADPH respectively in order to reduce 3-PGA to triose phosphates (G3P). Finally, the regeneration phase, composed by several enzymatic steps, uses triose phosphates (G3P) and ATP in order to regenerate RuBP. The entire cycle starts with the activity of ribulose-1,5-bisphosphate carboxylase/oxygenase (RuBisCO) and is closed with the activity of phosphoribulokinase (PRK). It uses the energy delivered by the thylakoid reactions to synthesize one molecule of G3P for every three cycles of CO_2_ assimilation. G3P serves as building block for biomass or as energy carrier. It is noteworthy that every biological cycle inherently suffers from slips and leaks and has to be maintained by influx of electrons and/or cofactors (Nelson et al., 2002).

In eukaryotic photosynthetic organisms (i.e., algae and plants), the entire photosynthetic process occurs in a highly specialized enclosed compartment, the chloroplast. The chloroplast has originated from an endosymbiotic event where a photosynthetic bacterium was engulfed by a host eukaryotic cell (Marín-Navarro et al., 2007; Zoschke and Bock, 2018). Then, a massive gene transfer occurred between the chloroplast and the nucleus and the chloroplast function evolved to specialize on photosynthesis. Most of the chloroplast localized proteins are nuclear encoded. However, 80–150 photosynthetic proteins remain encoded in the chloroplast genome. Among these proteins are notably subunits of PET-chain complexes and of Rubisco. It is believed that a chloroplast localized expression of these genes facilitates an efficient regulation of photosynthesis. In accordance with the chloroplast’s origin, its transcription and translational machinery differs from the nuclear one and resembles the bacterial one (Zoschke and Bock, 2018).

An important part of the research pursuing a better understanding of photosynthesis was done by studying mutants with defective photosynthesis (Levine, 1969; Somerville, 1986; Dent et al., 2001). The green alga *Chlamydomonas reinhardtii* has served as an important model organism to study photosynthesis (Rochaix, 1995). A great advantage of using *C. reinhardtii* for studying photosynthetic mutants has been that it can grow either prototropically or heterotrophically. Respectively, many photosynthesis deficient mutants of *C. reinhardtii* have been isolated by identifying their inability to grow photoautotrophically (Dent et al., 2001). There are two types of photoautotrophic mutants. In the first type, chloroplast development is arrested at some stage, causing gross structural abnormalities, which inhibit photosynthesis. In the second type of mutants, most of the biochemical apparatus of the chloroplast is present and functional, while only single components are defective. The defective component in these mutants could be an enzyme of the CBB-cycle, a carrier molecule of the electron transport chain or a factor to couple photosynthetic phosphorylation to electron transport (Levine, 1969). These elements are coupled with each other to form the photosynthetic apparatus. Therefore, a mutation that affects a single component may cause the inactivity of another. The study of photosynthetic mutants enables to entangle the complex relationships between photosynthetic components. However, mutants expressing their phenotype constitutively may only resolve the overall implications of their mutation. In order to differentiate between the direct and indirect effects of a mutation, a conditional expression of the phenotype comes in very handy. Several studies reported the isolation of mutants with temperature-sensitive photosynthesis (Weaver, 1971; Sherman and Cunningham, 1977; Lavintman et al., 1981; Spreitzer et al., 1988; Sarma and Singh, 1995; Liu et al., 2007). These mutants exhibited a defective photosynthetic machinery at high temperature. Their phenotype was characterized to varying extend. To the best of our knowledge, the genotype of only one of these mutants (68-4PP) has been identified and reported (Chen et al., 1988). 68-4PP was a mutant of *C. reinhardtii* and carries the single amino acid change L290F on the large subunit of rubisco (RbcL).

In an attempt to control photosynthetic activity to support continuous hydrogen production, we previously generated and isolated temperature-sensitive-photoautotrophic (TSP) mutants in *C. reinhardtii* (Bayro-Kaiser and Nelson, 2016). We randomly mutagenized cells by UV-exposure and identified relevant mutants that were NOT able to grow (photoautotrophically) at elevated temperature. This tedious “negative selection” yielded mutants that would have been otherwise neglected (Supek et al., 1996). 12,000 colonies grown from UV-exposed cells were screened, among which, 157 TSP mutants were identified. The average yield of 1 TSP mutant per 76 screened colonies was unexpectedly high. This suggested a high mutagenesis rate and that the TSP mutants carried a large number of mutations which were not causative for the TSP phenotype. Such silent mutations had to be cleared out by extensive crossing and further selection in order to identify the TSP relevant genotype. Thorough phenotyping of all TSP mutants enabled selecting the four most interesting mutants according to a variety of criteria (e.g., no abnormal phenotype at the permissive temperature, viability of partial photosynthetic reactions at restrictive temperature, capacity to recover from heat treatment). The selected mutants were called TSP1, TSP2, TSP3, and TSP4 and their relevance for hydrogen production was described in a previous report (Bayro-Kaiser and Nelson, 2016).

In order to further understand these mutants and their broader relevance to the study of photosynthesis, we genetically characterized them by crossing and whole genome sequencing. We identified single amino acid changes in the chloroplast elongation factor G (CEF-G), phosphoribulokinase (PRK) and an essential subunit of PSII (PsbO) in TSP1, TSP2 and TSP4 respectively. CEF-G is essential for the translation of chloroplast encoded proteins. PRK is essential for the CBB-cycle. While, PSII is a key component of the PET-chain. All three components are essential for photosynthesis. This report presents point mutations on components of three distinct and essential parts of the photosynthetic apparatus which resulted in temperature-sensitive photosynthesis. Furthermore, the implications and relevance of these mutants as tools for photosynthesis research are discussed.

## Methods

### Strains and Culturing

The mutants TSP1, TSP2, and TSP4 were produced on the background strain of C. reinhardtii 1A+ and were previously described (Bayro-Kaiser and Nelson, 2016). The mutants were deposited in the public mutant bank “www.chlamy.org” with the respective numbers CC-5656, CC-5657 and CC-5669. 1A+ and the mutants are of mating type (+). The C. reinhardtii wild type strains 7A- and S1D2 are of mating type (-) and were used for backcrossing and outcrossing respectively. The cells were grown on solid and liquid TAP medium (Stern et al., 2008) under continuous illumination of 50–70 µE m^-2^ s^-1^ and a temperature of 25°C. heat treatment was performed at 37°C.

### Immunoblotting

To prepare crude protein extracts, 1.5 ml of cell culture (OD_730 =_ 0.4–0.6) was centrifuged (2 min, 14,000 x g, room temperature) and the pellet was resuspended in Laemmli protein buffer (65.8 mM Tris-HCl, pH 6.8, 2.1% SDS, 26.3% (w/v) glycerol, 0.01% bromophenol blue). The final concentration corresponded to a chlorophyll content of 0.14 μg μl^-1^. Extracts were incubated at RT overnight, then centrifuged (2 min, 14,000 x g) to pellet the cell debris. Supernatants were loaded onto a sodium dodecyl sulfate polyacrylamide gel for electrophoresis (SDS-PAGE) to separate the proteins. The polyacrylamide gel comprised 5 and 16% acrylamide sections for protein collection and separation, respectively. The separated proteins were transferred to a nitrocellulose membrane with a semi-dry transfer cell (Trans-Blot SD from Bio-Rad), according to the manufacturer’s instructions. The membrane was incubated for 1 h at room temperature in blocking buffer (20 mM Tris-HCl, pH 7.4, 0.5 M NaCl, 0.1% Tween-20, 5% (w/v) skim milk powder), then overnight at 4°C in blocking buffer with a primary antibody, diluted according to the manufacturer’s recommendation (Agrisera). Primary antibodies were from rabbit against D1, PsbO, Cytf, PsaA, PsaC, AtpB, RbcL and PRK. The respective product numbers were AS11 1786, AS06 142-33, AS06 119, AS10 939, AS05 085-10, AS03 037, AS07 257. Next, the membrane was washed 3 times for 15 min in blocking buffer, then incubated for 1 h at room temperature in blocking buffer with the secondary antibody (peroxidase-conjugated goat-anti-rabbit IgG from Jackson ImmunoResearch), diluted 1:5,000. This was followed by a second wash repeated 3 times for 15 min with blocking buffer, then once for 15 min with blocking buffer that lacked powdered milk. The peroxidase substrate (Western lightning Plus-ECL from Perkin Elmer) was added to the secondary antibody, according to the manufacturer’s instructions. The luminescent signal was detected by an imager (Amersham Imager 600).

### gDNA Preparation

100 ml of liquid culture was centrifuged at 1,000 x g for 2 min and the pellet was washed in 6 ml of TEN buffer (10 mM Tris; 30 mM EDTA; 150 mM NaCl; pH 7.0). Centrifugation at 1,000 x g for 2 min was repeated. The pellet was resuspended in 1 ml of water and 2 ml SDS-EB buffer (0.1 M Tris; 50 mM EDTA; 0.4 M NaCl; 2% SDS; pH 8.0). 2 ml of phenol and 2 ml of chloroform-isoamyl alcohol (24:1) were added. The sample was gently mixed and centrifuged at 2,950 x g for 15 min. The upper phase was transferred into a new tube and incubated with 70 μl of RNase (Fermentas) for 2 h at 37°C. 2 ml of phenol and 2 ml of chloroform-isoamyl alcohol (24:1) were added, the sample was gently mixed, centrifuged at 2,950 x g for 15 min and the upper phase was transferred into a new tube. The last step was repeated without phenol. The upper phase was transferred into a new tube, 2 volumes of 100% ethanol were added and the mixture was incubated on ice for 30 min. The sample was centrifuged at 2,950 x g for 10 min, the pellet was washed in 1 ml of 70% ethanol and centrifugation was repeated. The pellet was dried at 37°C and resuspended in 50 μl of water.

### Crossing

The strains to be crossed were transferred to a N10 TAP plate (i.e., TAP medium with only 10% of the amount of NH_4_Cl). These plates were incubated at low light (10 μE m^-2^ s^-1^) for 3 days to induce gametogenesis. Afterwards, they were resuspended individually in 2.5 ml of sterile DDW in 15 ml conical tubes. These were incubated shaking for 2 h under high light intensity (500 μE m^-2^ s^-1^) to induce motility of the gametes. Afterwards, these suspensions were mixed pairwise. Each pair being two different strains of opposing mating type. The mix was further incubated without shaking under high light intensity (500 μE m^-2^ s^-1^). After an additional 2 h, 200 μl of the cell suspension were plated on one side of a fresh TAP agar plate. This was done carefully to not agitate the suspension and to not separate mating cell pairs. The plates were left at low light overnight and then maintained in total darkness at 25°C for zygospore maturation during one week. During this week, the maturating zygospores were covered by growing cells that did not form zygospores. These cells were scraped off the plate with a sterile scalpel. While, the zygospores being tightly stuck to the agar were not removed. These plates with exposed mature zygospores were incubated for 16 h in the light to induce zygote meiosis and the release of four progeny cells. These were dragged with a micromanipulator and placed on different locations of the agar plate. The plate was kept at 25°C in the light to grow the single cells to visible colonies. The colonies were replated on fresh agar plates and stocked. Progenies for which not all four sister cells survived, were discarded. When required, the mating type of progeny cells was determined by PCR as described elsewhere (Zamora et al., 2004).

### Sequencing and Data Analysis

The DNA libraries preparation and subsequent High-throughput sequencing was performed by the QB3 Vincent J. Coates Genomics Sequencing Laboratory. The size of the DNA fragments of the library preparations were of 300 – 700 bp. These were sequenced on an Illumina4000 sequencer. Sequencing of 100 bp from each end of the fragment was performed (i.e., 100 bp paired end sequencing). The output data obtained were tens of millions of 100 bp sequences. These data sets were analyzed with the CLC genomics workbench program.

### Complementation of TSP4

The entire PsbO sequence including 1,000 bp upstream were amplified by PCR from the background wild type strain 1A+. The obtained fragment was cloned into the psl18 plasmid by Gibson Assembly (New England Biolabs). The available promoter, 5’UTR and 3’UTR sequence available on the psl18 plasmid to express heterologous inserts was omitted since we used the entire native sequence of PsbO. The plasmid was overexpressed in E. coli and isolated with a mid-prep kit (Promega). Then, the plasmid was transformed into the TSP4 mutant by electroporation as described elsewhere (GeneArt Chlamydomonas, Invitrogene). The antibiotic containing selection plates (20 µM paromomycin) with fully grown transformant colonies were transferred to 37°C for 24 h. Then, the amount of PSII reaction centers (F_v_/F_m_) was measure on each colony by the use of a portable fluorometer (Junior-PAM). The colonies that presented some significant amount of PSII reaction centers were re-plated for further measurements on liquid medium.

### 3D Models

The models were obtained from the PDB protein data bank. The accession numbers were 4myt, 6h7g and 6kac for EF-G, PRK, and PsbO, respectively. The amino acid sequence was changed using Coot (Emsley et al., 2010) and image generation was done with PyMOL (The PyMOL Molecular Graphics System, Version 1.2r3pre, Schrödinger, LLC).

## Results

### Phenotype

**Figure 1:**
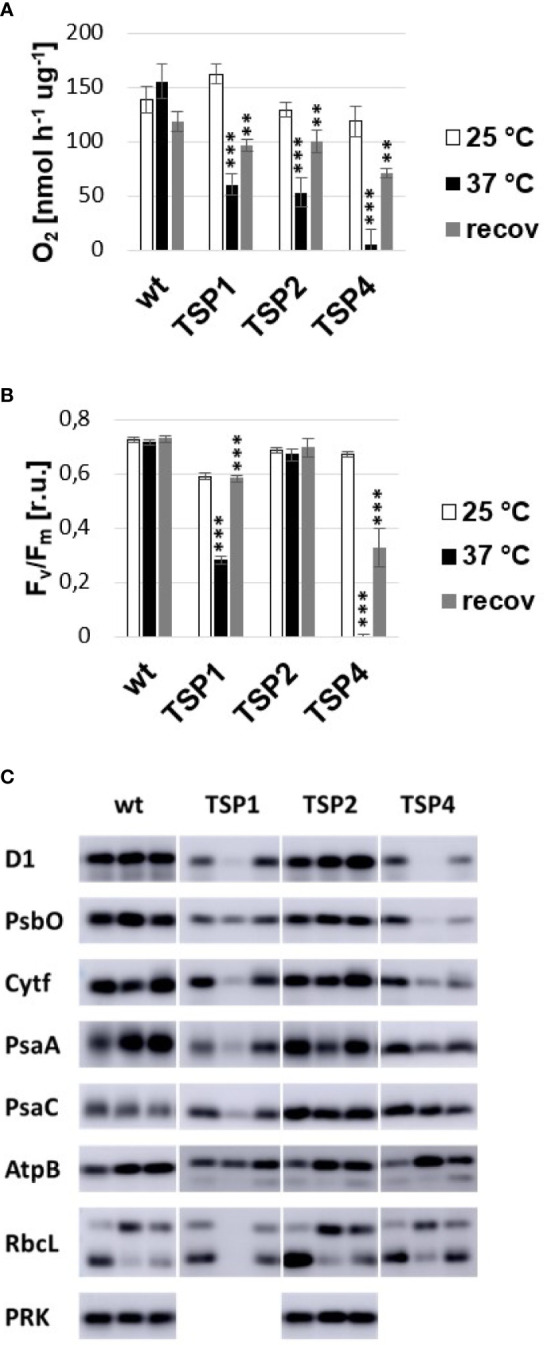
Phenotype of temperature-sensitive-photoautotrophic (TSP) mutants. **(A)** Light driven oxygen production. **(B)** Maximum quantum efficiency of PSII. **(C)** Immunoblotting against subunits of major photosynthetic complexes. Samples of wild type and TSP mutant cultures were analyzed at three conditions: during growth at 25°C, after adaptation to 37°C for 24 h and after subsequent recovery at 25°C for 24 h. The respective bands in **(C)** are presented from left to right. All immunoblots were repeated independently 2 to 4 times and the presented phenotype was confirmed. The presence of phosphoribulokinase (PRK) was tested only in the wild type and TSP2. The data presented in **(A, B)** corresponded to the average of 3 independent experiments and the respective standard deviation. Statistical analysis was performed by performing a paired t-test for “37°C vs 25°C” and “recov vs 37°C” for each strain. The significance level is indicated as follows: no sign means p > 0.05, **means p < 0.01 and ***means p<0.001.

At high temperature, PSII mediated oxygen production in TSP1, TSP2, and TSP4 was only 30, 30, and 0% compared to the wild type (**Figure 1A**). In mutants TSP1 and TSP4, it could be explained by a reduction of PSII reaction centers by 60% and 100% respectively upon high temperature treatment (**Figure 1B**). Although in mutant TSP2, the amount of PSII reaction centers remained unchanged by high temperature treatment. Upon this, the effect of temperature on the amount of a variety of subunits from all major photosynthetic complexes was determined by Immunoblotting: PSII (D1 and PsbO), Cytochrome b_6_f complex (Cytf), PSI (PsaA, PsaC), ATP-synthase (AtpB), Rubisco (RbcL), and Phosphoribulokinase (PRK) (**Figure 1C**). In mutant TSP1, D1, Cytf, PsaA, and RbcL were all drastically decreased at high temperature. PsbO was also reduced, although to a lesser extent, while, AtpB seemed to be unaffected. In mutant TSP2, the amount of none of the subunits was affected by temperature. In mutant TSP4, D1 was undetectable at high temperature, while PsbO was reduced dramatically. Cytf was considerably reduced while PsaA was slightly reduced. All other subunits were unaffected.

### Genotyping

#### Preliminary Whole Genome Sequencing

**Figure 2:**
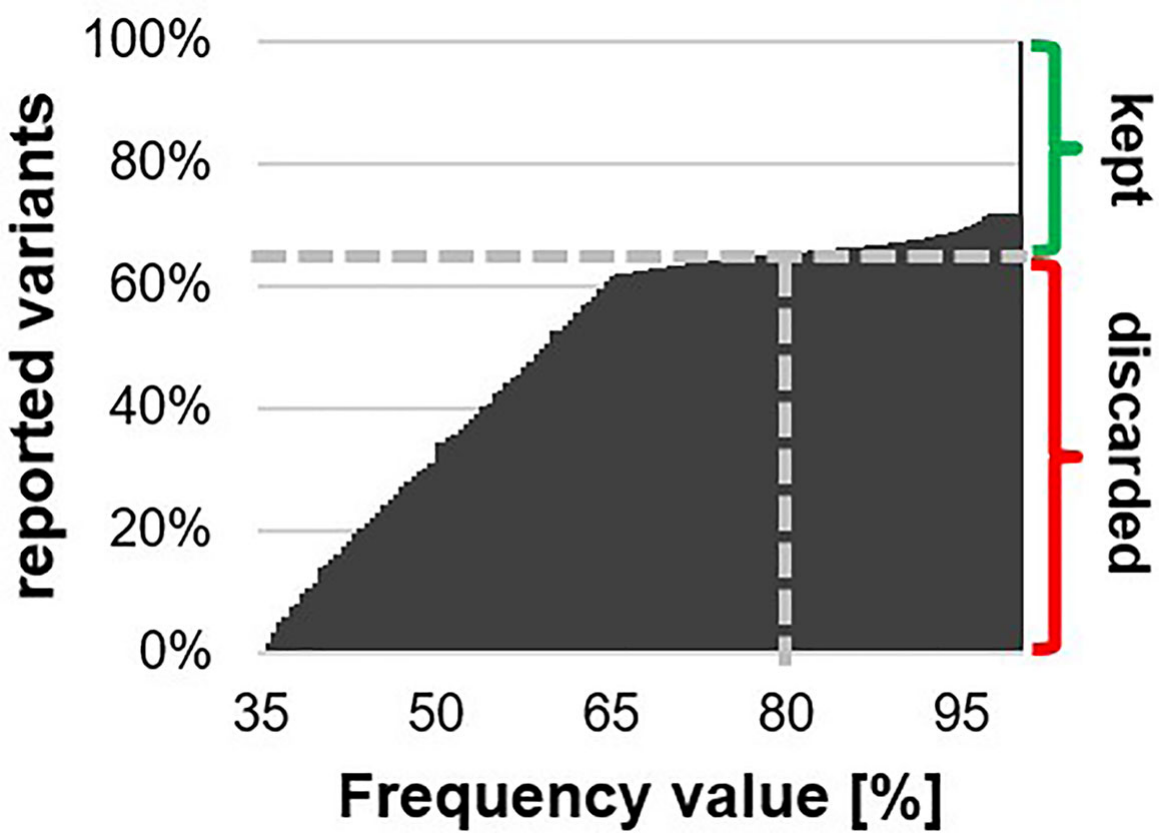
Cumulative histogram on all reported variants distributed by their respective frequency value. The grey dashed line indicates the fraction of reported variants that had a frequency value below 80%. These variants were discarded as falsely reported, while, the remaining variants were kept as potentially real.

Genomic DNA was extracted from wild type and mutant cultures (TSP1, TSP2, and other not reported mutants) grown at 25°C to the late logarithmic growth phase. The extracted gDNA was sent to the QB3 Vincent J. Coates Genomics Sequencing Laboratory. There, DNA libraries were prepared from the sent gDNA and sequenced on an Illumina4000 high throughput sequencer. The obtained data was sent back to us as millions of small 100 bp sequences (i.e., reads). Then, the sequencing data was processed with a genomics software tool (CLC workbench). The reads for each strain were mapped onto the full sequence of the online available *C. reinhardtii* genome (phytozome). An average coverage of 20 was achieved, meaning that on each nucleotide of the genome sequence an average of 20 reads were mapped. Then, a variant detection analysis was performed, which identified variants (mainly SNPs) in the mapped reads. Each reported variant was associated to a frequency value. This frequency value indicates which percentage of the reads that were mapped to the respective nucleotides, reported the respective variant. Since C. reinhardtii is a haploid, we would have expected the frequency of each reported variant to be 100%. However, artificially generated variants could have arisen during any step between gDNA isolation and data processing (Ma et al., 2019). Accordingly, there was a large bulk of reported variants with frequency values between 35 and 65%. For the wild type, 62% of all called variants had frequency values between 35 and 65%, while 28% of them had a frequency value of exactly 100% (**Figure 2**). Conversely, ambiguously called bases during sequencing may have resulted in single reads failing to report a real variant. To account for low quality base calling, we decided to set a safe cutoff of 80%. We kept all variants with a frequency value over 80%. The variants reported in each mutant were filtered against the variants reported in our background wild type. The remaining variants were filtered for non-synonymous variants. 60 and 55 unique non-synonymous variants distributed among all chromosomes were reported in mutants TSP1 and TSP2 respectively. These mutants were generated by random UV induced mutagenesis. Therefore, while these lists of reported variants may have still contained falsely reported variants, they may have also contained real mutant variants that were not causative for the phenotype of interest (i.e., TSP phenotype).

#### Preliminary Mapping by Crossing

*C. reinhardtii* has the ability to reproduce sexually. When two opposing mating types of *C. reinhardtii* are crossed, they form diploid zygotes. These zygotes undergo meiosis and recombination to release 4 progeny cells with differential genotype. These cells can be separated from each other with a micromanipulator and grown into single colonies on an agar plate. The mutants (TSP1, TSP2, and TSP4) were backcrossed to the respective opposing mating type of the background wild type strain (7A-). Then, the resulting progenies were screened for a TSP phenotype. For each mutant, a progeny population resulting from 10 zygotes were screened. For all mutants, every zygote resulted in two progeny cells with a TSP phenotype and two progeny cells with no TSP phenotype. This suggested that the phenotype of each mutant was caused by a single mutated locus.

#### Outcross and Pooling Progenies by Phenotype

**Figure 3:**
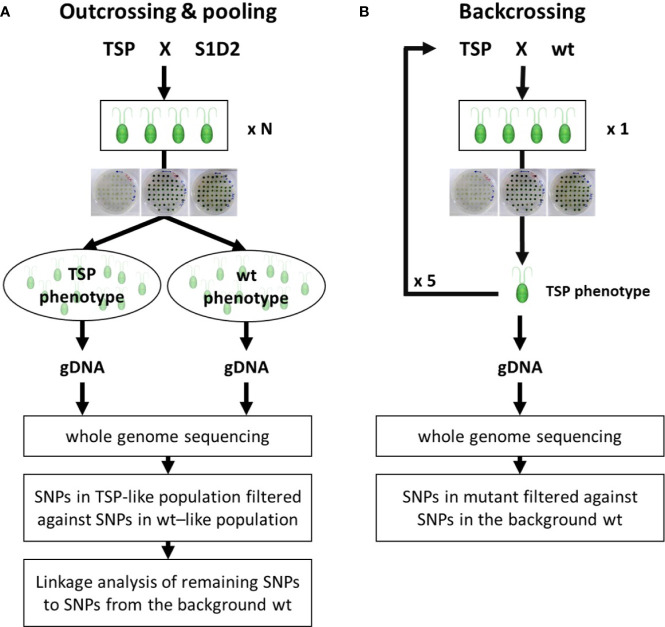
Flow chart of genotyping experiments. **(A)** Outcrossing & pooling. A temperature-sensitive-photoautotrophic (TSP) mutant was crossed with the outcross wild type strain S1D2. A large number of resulting tetrads (N=15 and N=25) were isolated and dissected. The respective progenies (60 and 100) were tested for a TSP phenotype and pooled according to it. A single gDNA sample for each pool was extracted and used for whole-genome sequencing and respective analysis. **(B)** Backcrossing. A TSP mutant was crossed with the background wild type strain. A single resulting tetrad was isolated and dissected. The four progeny cells were tested for a TSP phenotype. A single progeny with TSP phenotype was selected and crossed again to the background wild type. The procedure was repeated 5 times before isolating gDNA for whole-genome sequencing and respective analysis.

Outcrossing helped to differentiate between falsely reported mutant variants and real mutant variants. While, pooling progenies by their phenotype helped distinguishing between real variants that were causative and real variants that were not causative for a TSP phenotype (**Figure 3A**). Outcrossing consists of crossing a mutant strain to a different wild type strain which carries many unique SNPs. As mutant variants are genetically linked to regions from the background wild type strain, real mutant variants in an outcross progeny should have in close vicinity mostly SNPs from the background wild type strain. Respectively, any reported variants that are in close vicinity of unique SNPs from the outcross wild type strain can be discarded as falsely reported variants. Pooling progenies by their phenotype consists of preparing and sequencing a single gDNA preparation from a large pool of progenies which were selected for a specific phenotype. Any real mutant variant that is causative for the respective phenotype, will have to be reported with a frequency value of 100%. While, real mutant variants that are not causative may be reported with frequencies below 100%, since some progeny may have replaced these variants by the wild type genotype without losing the mutant phenotype of interest.

The mutants TSP2 and TSP4 were outcrossed to the wild type strain S1D2. Like in the previous experiment, many progeny cells of the cross were isolated and screened for a TSP phenotype. The progenies of 15 and 25 zygotes of the outcross for TSP2 and TSP4 respectively were screened. Then, these progeny strains were grown in liquid TAP at 25°C to the late logarithmic phase and pooled together according to their phenotype and progenitors. The pools were the following:

**1** - 30 progeny strains from TSP2 with TSP phenotype
**2** - 30 progeny strains from TSP2 with no TSP phenotype
**3** - 50 progeny strains from TSP4 with TSP phenotype
**4** - 50 progeny strains from TSP4 with no TSP phenotype

gDNA was extracted from each pool. Additionally, gDNA from the background strain 1A+ and the outcross strain S1D2 were extracted. These 6 gDNA preparations were sent to the previously mentioned genomics sequencing laboratory for DNA library preparations and subsequent high throughput sequencing. The obtained reads for each gDNA preparation were mapped on to the online available genome sequence as previously described. An average coverage of 30 was achieved. This was followed by a variant detection analysis. The sequencing data for each pool was obtained from a mix of genomes. Therefore, in contrast to the previous sequencing, we did expect variants with frequency values different than 100%. We expected the variants that caused a TSP phenotype to have a frequency of 100% in the pools number 1 and 3 (TSP phenotype). As previously done, we chose a cut off of 80% in order to account for ambiguously base calling. Conversely, we expected that any variant with any frequency value in the pools number 2 and 4 (wt phenotype) could not cause a TSP phenotype. This way, the variants reported in pools 1 and 3 (with frequency values > 80%) were filtered against the variants reported in pools 2 and 4 (with any frequency value). Additionally, they were filtered against the variants found in both wild type strains and selected for non-synonymous variants. A total of 21 and 20 nonsynonymous mutant variants remained for TSP2 and TSP4 respectively. A linkage analysis of these variants with wild type SNPs was performed. The TRIO tool of the CLC workbench program was performed on the full list of reported variants for pool number 1 and 3. This way, each reported variant was tagged with its respective origin: background wild type, outcross wild type or novel (i.e., mutant variant). The regions flanking the previously selected 21 and 20 mutant variants were searched for S1D2 SNPs. The sizes of the flanking regions free of S1D2 SNPs were determined. For mutant TSP2, there was one large region of 3 Mbp in chromosome 12 which had only mutant and 1A+ variants. This region contained 1 out of the 21 selected variants. All other regions were of only few thousands of base pairs. In mutant TSP4, two large regions of 3.1 and 1.8 Mbp were identified with only mutant and 1A+ variants. Both regions were on chromosome nine and in close vicinity. They contained 4 out of the 20 selected mutant variants. All the other analyzed regions were of a few thousands of base pairs.

#### Backcross

Meanwhile, all three TSP mutants were being backcrossed to the background wild type strain (**Figure 3B**). Backcrossing and subsequent screening for a TSP phenotype generated progeny strains which inherited the mutant variants which caused the TSP phenotype. However, they did not necessarily inherit the mutant variants which did not cause the TSP phenotype. Like previously described, each mutant was crossed with the opposing mating type of the wild type strain 1A+. The progeny was screened for a TSP phenotype and their mating type was determined by PCR. A progeny strain with a TSP phenotype and a mating type (-) was selected for each mutant. This first-generation of progeny was crossed with 1A+ and the same selection process was performed to obtain a second-generation progeny with a TSP phenotype and a mating type (-). This process was iterated until a fifth-generation with a TSP phenotype was obtained. gDNA was extracted from these strains and whole genome sequencing and mutant variant identification was performed as described in the preliminary whole genome sequencing experiment. This time, only 6, 3, and 5 mutant non-synonymous variants were reported for TSP2, TSP3, and TSP4, respectively.

### TSP1

**Figure 4:**
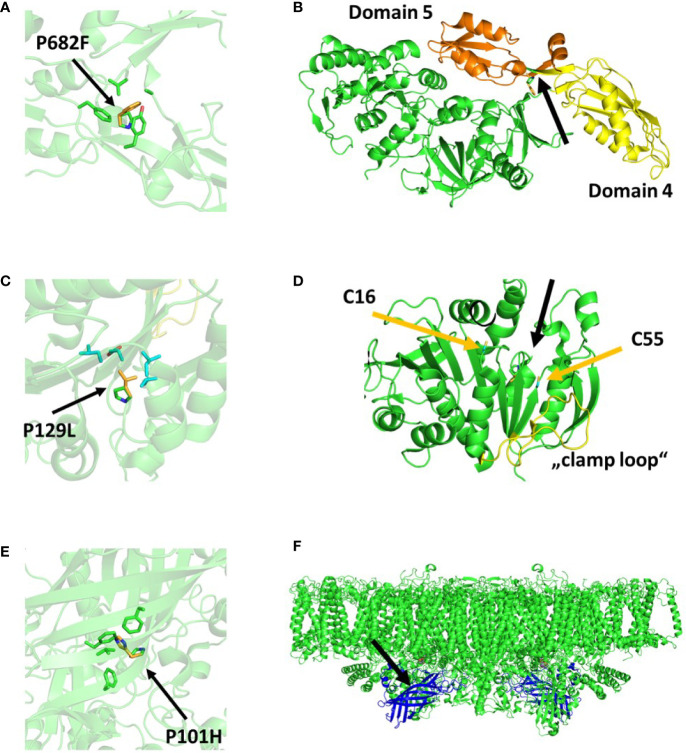
Protein structural models displaying the consequences of the respective mutations in TSP1 **(A, B)**, TSP2 **(C, D)**, and TSP4 **(E**, **F)**. **(A, C, E)** display a zoom in view where the single amino acid change is indicated (green=native, yellow=mutant) as well as the surrounding amino acids. **(B, D, F)** display a zoom out view where the mutation site is indicated with a black arrow. In **(B)**, domains 4 and 5 of EF-G are colored yellow and orange respectively. In **(D)**, the cysteines forming a disulfide bond when Phosphoribulokinase (PRK) is inactive are indicated with yellow arrows. The relaxed region facilitating activation of PRK (“clamp loop”) is colored yellow. In **(F)**, Photosystem II (PsbO) is shown in blue assembled into a PSII dimer complex. The models were obtained from the PDB protein data bank and their accession numbers are 4myt **(A, B)**, 6h7g **(C, D)**, and 6kac **(E**, **F)**, respectively.

For mutant TSP1, the preliminary sequencing and backcrossing experiments were performed. Respectively 60 and 6 non-synonymous mutant variants were reported. Between both experiments, there was only one commonly reported variant. The respective variant site was amplified by PCR in both the mutant and the wild type and their respective sequence was determined by sanger sequencing. The presence of the reported mutation was confirmed exclusively in TSP1. This variant (Cre02.g076250.t1.1:c.2044_2045delCCinsTT) was a MNP (GG to AA) within the coding sequence of the Chloroplast Elongation Factor G (CEF-G) which resulted in the single amino acid change Pro682Phe. There was no available structural model for CEF-G from *C. reinhardtii*. However, a high homology with the bacterial elongation factor G permited us to visualize the analog mutation in the available model of the respective homolog in *T. thermophilus* (**Figure 4A**). A sequence alignment resulted in 56.91% amino acid identity and 100% amino acid identity at the mutation site and surrounding amino acids (i.e., 680–684). The respective amino acid change replaced a small side chain (Pro) by a larger bulky side chain (Phe) in a tight hydrophobic pocket composed by two tyrosine and a phenylalanine. The mutation site was connecting domains 4 and 5 of the EF-G (**Figure 4B**). This change would have required a change of the secondary structure surrounding the mutation site.

### TSP2

For mutant TSP2, all three sequencing experiments were performed. Respectively 55, 1 and 3 non-synonymous mutant variants were reported. The single variant that was reported in the outcrossing experiment was as well reported in the other two experiments. As well noteworthy, this variant was the only commonly reported variant between the experiments that reported more than 1 variant. The respective variant site was amplified by PCR in both the mutant and the wild type and their sequence was determined by sanger sequencing. The presence of the reported mutation was confirmed exclusively in TSP2. This variant (Cre12.g554800.t1.2:c.479C>T) was a SNP (G to A) within the coding sequence of the Phosphoribulokinase (PRK) which resulted in the single amino acid change Pro160Leu (Pro129Leu in the mature PRK) (**Figure 4C**). The change occurred in close vicinity of Cys16 and Cys55 which was known to be crucial for the activation of PRK (**Figure 4D**). While the activity of PRK might have been reduced at high temperature, the amount of the protein was unchanged upon incubation at high temperature (**Figure 1C**).

### TSP4

**Figure 5:**
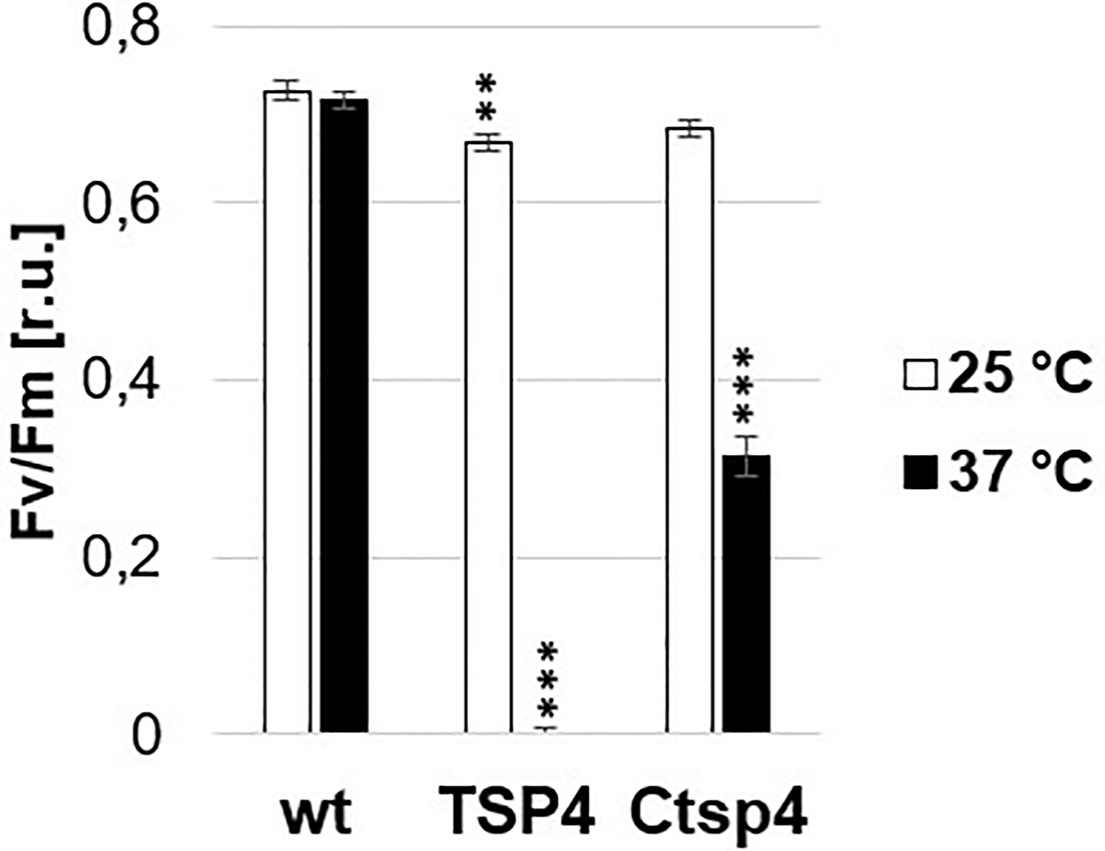
Phenotype of a TSP4 strain complemented with the native PsbO gene. The chart corresponds to the maximum quantum efficiency of PSII in wild type, TSP4 and Ctsp4 (complemented strain) cultures under two conditions: during growth at 25°C and after adaptation to 37°C for 24 h. The data corresponds to the average of three independent experiments and the respective standard deviation. Statistical analysis was performed by performing an unpaired t-test for “TSP4 vs wt” and “Ctsp4 vs TSP4” for each temperature. The significance level is indicated as follows: no sign means p > 0.05, **means p < 0.01 and ***means p < 0.001.

For mutant TSP4, the outcrossing and backcrossing experiments were performed. Respectively 4 and 5 non-synonymous mutant variants were reported. Between both experiments, there were 2 commonly reported variants. Both variant sites were amplified by PCR in both the mutant and the wild type and the respective sequences were determined by sanger sequencing. The presence of both reported mutations was confirmed exclusively in TSP4. The first variant (Cre09.g396213.t1.1:c.302C>A) was a SNP (C to A) within the coding sequence of PsbO. While, the second SNP (Cre09.g390150.t1.1:c.296T>A) was on a hypothetical gene. Both SNPs were on chromosome 9 and about 2Mbp apart from each other. In the outcrossing experiment, these two SNPs were reported with frequency values of 100 and 88%, respectively. The SNP on PsbO resulted in the single amino acid change Pro101His. The change resulted in the replacement of a small non-polar side chain (Pro) for a large bulky positively charged side chain (His) in a hydrophobic pocket surrounded by three large non-polar phenylalanine (**Figure 4E**). This change occurred in the center of the beta barrel domain of PsbO and would have required a significant change of the secondary structure surrounding the mutation site (**Figure 4F**). Subsequentially, TSP4 was transformed with a plasmid construct containing the full wild type PsbO gene (including 1,000 bp upstream) and an antibiotic resistance gene (against paromomycin). The obtained transformants were screened for PSII fluorescence at high temperature. One transformant was identified with a relatively high PSII fluorescence at high temperature (**Figure 5**). This transformant reduced PSII fluorescence down to only 40% after 24 h of heat treatment, while in TSP4, PSII in undetectable. The PSII fluorescence level remained unchanged in the complemented strain after additional 24 and 48 h of heat treatment.

## Discussion

### Sequencing Analysis

Whole genome sequencing is prone to a variety of errors (Ma et al., 2019). Any artificially introduced mutation, during any step from DNA isolation to sequencing, will result in false positively reported mutations with reported frequency values ranging from 0 to 100%. False positively reported mutations are highly probable. On the contrary, false negatives are highly improbable since they can arise only if a real mutation is replaced by the exact wild type polymorphism. Although, an ambiguous base call, caused by low quality base detection, can result in failing to report a real mutation in that specific read. Therefore, we can expect that the reported frequency values for real mutations may be somewhat lower, however not significantly lower, than 100%. Therefore, we considered any mutation with frequency values above 80%. This enabled us to discard the great majority of false positively reported mutations while it allowed ambiguous mutant bases in up to 20% of the respective reads. While, comparing two independent sequencing experiments enabled us to discard remaining false positively reported mutations.

### TSP1

After 5 times backcrossing, we were able to clean TSP1 from all non-synonymous mutations which are not causative for the TSP phenotype. Respectively, the 5th backcross generation had only one real non-synonymous mutation. This mutation caused the single amino acid change Pro682Phe in the CEF-G which resulted in the respective TSP phenotype of TSP1. CEF-G is required for the elongation of chloroplast encoded proteins. All photosynthetic complexes of the PET-chain as well as Rubisco have some of their subunits encoded in the chloroplasts. Respectively, a defective chloroplast translation machinery would eventually lead to the reduction in the amount of those complexes. This has been clearly established by immunoblotting TSP1 samples after heat treatment. Subunits of PSII, Cytb6f complex, PSI, and Rubisco were reduced upon high temperature treatment. Only ATP synthase seemed to be unaffected. The chloroplast translation machinery is very much alike the bacterial translation machinery since it has been inherited from bacteria during an endosymbiotic event that gave rise to the chloroplasts (Beligni et al., 2004; Atkinson and Baldauf, 2011). Respectively, we analyzed the effect of the mutation on the available 3D model of the ortholog enzyme in *T. thermophilus*. Elongation factor-G is bound to the ribosome and catalyzes the translocation step during translation (Wintermeyer et al., 2011; Peng et al., 2019). It has 5 domains, were domains 4 and 5 are essential for translocation (Savelsbergh et al., 2000). A rotation of domain 4, respective to the rest of the enzyme, enables translocation. While domain 5 is thought to be involved in inducing the rotation of domain 4. The mutation site in TSP1 was found on the beta strand which connects domain 4 with 5. The mutation site itself and the surroundings are well conserved between the algal and bacterial protein. The respective amino acid change required a re-conformation of the surrounding secondary structure which may have resulted in a change of the interaction between domain 4 and 5. This suggested, that the mutation in TSP1 altered the protein conformation in such way that the normal interaction between these domains was hindered at high temperature.

### TSP2

After 5 times backcrossing, we were able to clean TSP2 from all non-synonymous mutations which are not causative for the TSP phenotype. Respectively, the 5th backcross generation had only one real non-synonymous mutation. This mutation caused the single amino acid change Pro160Leu in PRK which resulted in the respective TSP phenotype of TSP2. This conclusion was confirmed as well by the outcrossing experiment. The PRK catalyzes the phosphorylation of ribulose 5-phosphate by the use of ATP. Its activity is an essential step of the CBB-cycle. A *C. reinhardtii* mutant lacking PRK activity has been shown to have an intact PET-chain, however, a reduced amount of O_2_ production (Moll and Levine, 1970; Neumann and Levine, 1971). A non-functional CBB-cycle results in depletion of ADP and NADP+. As a result, the PET chain is slowed down and there are less available electron acceptors for PSII activity. The phenotype of TSP2 at high temperature agreed with the phenotype of a mutant lacking PRK activity. However, immunoblotting of TSP2 samples demonstrated that the amount of PRK was not affected by temperature treatment. Therefore, the mutation in TSP2 affected the activity of PRK at high temperature but it did not affect its expression or stability. The respective mutation was in close vicinity of Cys16, Cys55, and the flexible “clamp loop” region between them. These cysteines can form a disulfide bridge causing conformational changes in PRK. Oxidation and reduction of this bridge is mediated by thioredoxin and results in deactivation and activation of PRK respectively. The flexibility of the “clamp loop” is thought to facilitate these conformational changes (Thieulin-Pardo et al., 2015; Gurrieri et al., 2019). The mutation in TSP2 may have interacted with this mechanism and interfered with the activation of PRK at high temperature.

### TSP4

In TSP4, two mutations have been identified as possible causative mutations for the respective TSP phenotype. Both mutations were reported in the outcross experiment and both mutations were found in the 5th backcross generation of TSP4. Although on the outcross experiment, one mutation had a frequency value of 100% while the other had a frequency value of 88%. Furthermore, both mutations were on chromosome 9 and were separated by about 2 Mbp. This suggested that the mutation with a frequency value of 100% was the only causative mutation while the other mutation was non-causative but had a high probability of co-inheritance due to genetic vicinity. Consequently, the non-causative mutation could have prevailed throughout 5 rounds of backcrossing and selection as well as achieved a high prevalence (88%) in the outcross progeny which had a TSP phenotype. This suggestion was in concordance with the nature of the mutated genes. The mutation with a frequency value of 100% caused a single amino acid change in PsbO while the other mutation was on a hypothetical gene with no assigned function. PsbO is one of the extrinsic subunits of the PSII complex. It stabilizes the manganese cluster and its absence results in the degradation of the entire PSII complex (Pigolev and Klimov, 2015). It is an intrinsically disordered protein which was thought to undergo conformational changes along temperature changes (Popelkova and Yocum, 2011; Offenbacher et al., 2013). It has been shown that heat stress on spinach thylakoids resulted in the release of all extrinsic proteins from PSII and concomitant D1 cleavage and loss of PSII activity (Komayama et al., 2007). Respectively, increased tolerance to heat inactivation of spinach PSII was achieved *in vitro* by replacing its mesophilic PsbO by a PsbO from a thermophilic cyanobacterium (Pueyo et al., 2002). All this suggested, that mutations in PsbO could result in lower thermotolerance of PSII. Respectively, TSP4 has been demonstrated to degrade PSII completely upon high temperature treatment. Furthermore, PSII stability at high temperature in TSP4 has been partially recovered by the addition of the native wild type psbO gene into the TSP4 genome. The identified mutation on the psbO gene caused the single amino acid change Pro101His in PsbO which resulted in a temperature sensitive PSII. This is a significant change in the beta barrel domain of PsbO which could have altered the thermostabilizing properties of PsbO. As a result, high temperature treatment prompted the release of PsbO from the PSII complex and caused the entire complex to destabilize and degrade.

### Temperature Sensitive Photosynthesis

Temperature sensitive (Ts) mutants have been a powerful tool to study many essential proteins from diverse organisms (McMurray, 2014). It provided very valuable information on protein function and oligomer assembly. Recognition of the value of such mutants prompted the construction of Ts mutant banks (Li et al., 2011) and the development of methods for rational design of Ts proteins (Zeidler et al., 2004; Tan et al., 2014). These advancements have enabled many novel biological discoveries and Ts mutants are presently widely used (Tsepal, 2020). Yet, this powerful tool has hardly been exploited for the study of essential photosynthetic proteins. To the best of our knowledge, only two Ts photosynthetic mutants have been reported with a fully described genotype. A mutant with a Ts rubisco (68-4PP) was isolated by forward genetics (Chen et al., 1988) and a mutant with Ts translation of PetD was engineered by reverse genetics (Chen X. et al., 1993). Both mutants were in *C. reinhardtii* and both mutated genes were chloroplast encoded. 68-4PP had an unstable rubisco which degraded when exposed to high temperature. It was used to gain insights into the stability of Rubisco and on the interface and interaction between its large and small subunits (Chen Z. et al., 1993; Du et al., 2000; Spreitzer et al., 2005). TSP1, TSP2, and TSP4 carried mutations on nuclear encoded and chloroplast localized proteins. All three proteins are essential for photosynthesis and make up part of three different processes; chloroplast protein biogenesis, photosynthetic electron transport, and CBB-cycle. TSP1 can be used to further understand the translocation mechanism and might shed light on the required interaction between domains 4 and 5 of the CEF-G. Furthermore, TSP1 is an excellent platform to study the *de novo* biogenesis and assembly of major photosynthetic complexes. Biogenesis of these complexes has been much studied but many questions still remain unanswered (Rühle and Leister, 2016; Wittenberg et al., 2017). TSP2 can be used to further understand the thioredoxin mediated activation of PRK. Furthermore, it may facilitate a quick resumption of CBB-cycle activity after a period of inactivation since the mutated PRK is not degraded by high temperature. This can be a powerful tool to study photosynthetic metabolic fluxes and support kinetic modeling of the CBB-cycle (Janasch et al., 2018). The further study of TSP4 can broaden the understanding on the role of intrinsically disordered proteins as stabilizers of large complexes (Uversky, 2015). It is as well an excellent platform to study the specific *de novo* biogenesis of PSII.

## Conclusions

Negative selection proved to be an excellent approach for discovering uncharted subjects. By this method, we isolated temperature-sensitive-photoautotrophic mutants with deficient photosynthesis at high temperature. Three of which, revealed to carry single amino acid changes in essential proteins for three distinct processes required for photosynthesis: Chloroplast expression machinery, PET-chain and CBB-cycle. The respective mutations in key positions are likely to alter the proteins in such way that they are functional at the permissive temperature, however, defective at the restrictive temperature. These mutants present themselves as excellent and novel tools for the study of a wide range of aspects relevant to photosynthesis research. The mutants will be publicly available at the *C. reinhardtii* mutant bank: chlamycollection.org

## Data Availability Statement

The datasets generated for this study can be found in Zenodo, doi 10.5281/zenodo.3952517, https://zenodo.org/record/3952517#.Xx_5Pp4zbsA.

## Author Contributions

VB-K performed the experiments and wrote the article. NN supervised the work.

## Funding

This work was supported by the Israel Science Foundation (ISF Grant No. 569/17), by the Joint UGC - ISF Research Grant no. 2716/17, and by the German-Israeli Foundation for Scientific Research and Development (GIF Grant no. G-1483-207./2018).

## Conflict of Interest

The authors declare that the research was conducted in the absence of any commercial or financial relationships that could be construed as a potential conflict of interest.
